# Front Vehicle Detection Algorithm for Smart Car Based on Improved SSD Model

**DOI:** 10.3390/s20164646

**Published:** 2020-08-18

**Authors:** Jingwei Cao, Chuanxue Song, Shixin Song, Silun Peng, Da Wang, Yulong Shao, Feng Xiao

**Affiliations:** 1 State Key Laboratory of Automotive Simulation and Control, Jilin University, Changchun 130022, China; caojw18@mails.jlu.edu.cn (J.C.); scx@jlu.edu.cn (C.S.); pengsilun@jlu.edu.cn (S.P.); wangda_gspeed@jlu.edu.cn (D.W.); 2 School of Mechanical and Aerospace Engineering, Jilin University, Changchun 130022, China; songshx202@126.com; 3 Taizhou Automobile Power Transmission Research Institute, Jilin University, Taizhou 225322, China; 4 Zhengzhou Yutong Bus Co., Ltd., Zhengzhou 450016, China; SYL20081243@126.com

**Keywords:** computer vision, autonomous vehicle, SSD, deep learning, vehicle detection

## Abstract

Vehicle detection is an indispensable part of environmental perception technology for smart cars. Aiming at the issues that conventional vehicle detection can be easily restricted by environmental conditions and cannot have accuracy and real-time performance, this article proposes a front vehicle detection algorithm for smart car based on improved SSD model. Single shot multibox detector (SSD) is one of the current mainstream object detection frameworks based on deep learning. This work first briefly introduces the SSD network model and analyzes and summarizes its problems and shortcomings in vehicle detection. Then, targeted improvements are performed to the SSD network model, including major advancements to the basic structure of the SSD model, the use of weighted mask in network training, and enhancement to the loss function. Finally, vehicle detection experiments are carried out on the basis of the KITTI vision benchmark suite and self-made vehicle dataset to observe the algorithm performance in different complicated environments and weather conditions. The test results based on the KITTI dataset show that the mAP value reaches 92.18%, and the average processing time per frame is 15 ms. Compared with the existing deep learning-based detection methods, the proposed algorithm can obtain accuracy and real-time performance simultaneously. Meanwhile, the algorithm has excellent robustness and environmental adaptability for complicated traffic environments and anti-jamming capabilities for bad weather conditions. These factors are of great significance to ensure the accurate and efficient operation of smart cars in real traffic scenarios and are beneficial to vastly reduce the incidence of traffic accidents and fully protect people’s lives and property.

## 1. Introduction

Automobiles have become an indispensable and commonly used means of transportation for many families because of their huge traffic convenience, with the rapid development of the global economy and the gradual improvement of people’s living standards. However, the continuous growth of car ownership has also brought a series of traffic safety issues, thereby seriously affecting people’s quality of life and hindering the further development of society. Studies have shown that in an emergency, if the driver can be reminded to take effective driving manipulation one second in advance, then 90% of road traffic accidents can be avoided [1,2,3,4]. Many experts and scholars have turned their attention to smart cars with the vigorous development of modern information and automotive technologies. Considering that smart cars can autonomously drive, they have become an important way to eliminate hidden dangers of traffic safety [5,6]. Vehicle detection is an indispensable part of environmental perception technology for smart cars. Whether the front vehicle can be accurately detected is closely related to whether the autonomous vehicle can safely and steadily run. Intelligent vehicles can automatically identify the front vehicle objects on the basis of the vehicle detection technology by taking advantage of vehicle-mounted cameras. The driving vehicle can achieve important functions, such as vehicle distance maintenance, safe lane change, and collision warning, through timely and efficient feedback to the driver, which is conducive to prevent the occurrence of major traffic accidents and fully protect people’s lives and property safety [7,8,9]. Therefore, an in-depth study of such concepts is of great significance.

Front vehicles are the road obstacles in the actual driving environment, and vehicle detection refers to automatically detecting the vehicle in front from the collected pictures or video streams and making proper positioning. In the actual road scenes, bad weather conditions, complicated traffic environments, varying degrees of object occlusion, and differences in the vehicle characteristics make vehicle detection a challenging task [10,11,12]. Experts and scholars worldwide have shown increasing interest in the research of vehicle detection, with the development and improvement of intelligent transportation system and computer vision technology. Vehicle detection research methods are mainly divided into three types according to the different detection principles: feature-based, conventional machine learning-based, and deep learning-based detection methods. The feature-based detection methods mainly realize vehicle detection on the basis of the salient appearance features of the front vehicle. The common salient features include color, edge, symmetrical, and bottom shadow features of the vehicle. Teoh et al. [13] selected candidate regions of the front vehicle from the detection image on the basis of the symmetrical features of the vehicle. They also enhanced the edges of the regions and finally sent them to the support vector machine (SVM) classifier for verification. However, this method was easily affected by the viewing angle and natural environment; thus, it can only be applied to specific road scenes. Wang et al. [14] adopted an adaptive threshold segmentation algorithm to extract vehicle shadow features, used special masks to obtain vehicle object features at different distances, and combined with vanishing point constraints to achieve fast detection of front vehicles. He et al. [15] proposed a vehicle detection method around computer vision, which comprehensively utilized a variety of salient appearance features, such as edge, bottom shadow, and symmetrical features, and the detection precision was relatively high. The feature-based detection methods have low algorithm complexity and high detection efficiency in specific simple environments. However, the detection performance will be evidently reduced, and the environment adaptability will be poor when the road environment becomes complicated [16,17,18].

The conventional machine learning-based detection methods mainly use the feature description operator to extract the vehicle features and adopt the machine learning algorithm to train the samples. These methods select the appropriate feature classifier to realize vehicle detection. Zhang et al. [19] efficiently combined the multidimensional Haar-like features and Adaboost algorithm, and used a self-adaptive sky segmentation algorithm to segment the color space and multi-scale sub-window to scan the image in parallel. This approach effectively improved the efficiency of vehicle detection. Kim et al. [20] adopted a histogram of oriented gradients (HOG) on the basis of the position and intensity combined with a search space reduction method, which effectively reduced the overall calculation and sped up vehicle detection. Neumann et al. [21] used a stereo vision image classifier for vehicle detection on the basis of Haar-like, LBP (local binary patterns), and HOG fusion features and achieved good detection effects. The conventional machine learning-based detection methods do not have to rely on the prior knowledge of vehicle objects but still need the help of artificially designed image features. These methods cannot be well applied to vehicle detection with multiple working conditions and objects, and their generalization ability is poor [22,23,24].

In recent years, deep learning represented by convolutional neural network (CNN) has become an emerging development direction for machine learning with the rapid development of artificial intelligence technology and deep learning algorithms. This mechanism has achieved fruitful application results in image classification, speech recognition, and natural language processing. The deep learning-based detection methods mainly use deep convolutional neural networks to automatically extract vehicle object features and finally complete the vehicle detection task after classification. Lange et al. [25] adopted a 2D image vehicle detection system by using the depth information of LiDAR sensors to effectively shorten the calculation time of the algorithm, and finally obtained high detection precision through network topology optimization. Qu et al. [26] proposed a vehicle detection method on the basis of multi-scale spatial pyramid pooling (SPP), which can learn the characteristics of input images of different sizes. Liu et al. [27] proposed a two-stage detector for tiny vehicle detection. In the first stage, a backward feature enhancement network was used to generate high-quality region proposals. In the second stage, the spatial layouts of features of the region of interest (ROI) were obtained through the spatial layout preserving network. The experimental results showed that this method was helpful in obtaining a high recall rate and performed well in terms of detection precision. At present, the deep learning-based detection methods are mainly composed of two-stage and one-stage detection methods. Two-stage detection networks represented by Fast R-CNN, Faster R-CNN, and Mask R-CNN, generally have high detection precision. However, the algorithms based on region proposals often have high complexity and long calculation time, which cannot meet the real-time requirements of vehicle detection in the actual road scenes [28,29,30]. One-stage detection network is represented by YOLO, YOLOv2, and SSD. Although the detection speed has been significantly improved, the detection precision is not as good as the two-stage detection network. The deep learning-based detection methods are prone to “care for this and lose that”, and they still cannot simultaneously obtain good detection precision and detection speed [31,32,33]. All in all, the above three types of research methods have different drawbacks and disadvantages. The feature-based detection methods are easily restricted by environmental conditions, and the robustness is insufficient. The conventional machine learning-based detection methods have high manual dependence and poor generalization ability. The existing deep learning-based detection methods cannot balance accuracy and real-time performance. Therefore, this paper aims to improve the vehicle detection algorithm to obtain an ideal solution, so that the proposed algorithm can not only have good robustness and generalization ability in complicated environments and working conditions, but also achieve fast and accurate automatic vehicle detection.

In this research, a front vehicle detection algorithm for smart car based on improved SSD model is proposed. First, the SSD network model is briefly introduced, and its problems and shortcomings in vehicle detection are analyzed and summarized. Then, targeted improvements are performed to the SSD network model, including major advancements to the basic structure of the SSD model, the use of weighted mask in network training, and enhancement to the loss function. Finally, vehicle detection experiments are carried out on the basis of the KITTI vision benchmark suite and self-made vehicle dataset to observe the algorithm performance in different complicated environments and weather conditions. The proposed algorithm is comprehensively analyzed and evaluated by comparing the performance with the existing detection algorithms.

The remaining parts of this article are organized as follows: Section 2 determines the shortcomings of SSD in vehicle detection. Section 3 initiates targeted improvements to SSD. Section 4 conducts vehicle detection experiments by using appropriate datasets and observes and discusses the algorithm performance. Section 5 summarizes the conclusions and provides the possible work in the future.

## 2. SSD Network Model

### 2.1. Brief Introduction of SSD

SSD, which stands for “single shot multibox detector,” is one of the current mainstream object detection frameworks based on deep learning. SSD was originally raised by Wei Liu at the 14th European Conference on Computer Vision (ECCV) in 2016, and has become another one-stage object detection algorithm that attracted great attention after YOLO [34,35]. SSD not only draws on the anchor mechanism and feature pyramid structure of the Faster R-CNN, but also inherits the regression idea of YOLO and realizes the detection and classification of multiple bounding boxes on the basis of the simple end-to-end network. In comparison with Faster R-CNN, SSD does not require candidate region extraction, and the detection speed is faster. SSD does not use a fully-connected layer, and the detection precision is improved compared with YOLO.

**Figure 1:**
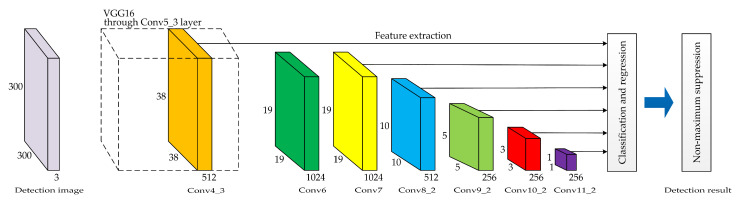
The basic structure of the SSD network model.

The SSD network model is mainly composed of three parts, including the basic network, feature extraction network, and detection network. The basic network is improved on the basis of VGG16 (visual geometry group 16). Considering that the fully-connected layer will interfere with the location information of the features, the last two fully-connected layers, namely, FC6 and FC7, are replaced by convolutional layers Conv6 and Conv7. Then, the following four sets of convolutional layers are added: Conv8, Conv9, Conv10, and Conv11. In each layer, 1 × 1 convolutional kernels are used for dimension reduction, and 3 × 3 convolutional kernels are utilized for feature extraction. Next, the feature maps of Conv4_3 and Conv7 are combined with those of Conv8_2, Conv9_2, Conv10_2, and Conv11_2 to form a multi-scale feature extraction network in the form of feature pyramids. Finally, two convolutional kernels with a size of 3 × 3 are used to perform convolutional operations on each feature map in the detection network. One convolutional kernel outputs category confidences, and the other provides the object location information for regression. All the calculation results are combined and transferred to the loss layer. The final detection result is outputted by using the non-maximum suppression (NMS) algorithm. Figure 1 shows the basic structure of the SSD network model, and Table 1 illustrates the main parameters of the SSD network model.

The SSD network model adopts multitask loss function, which mainly includes positioning and confidence errors. The total loss function is equal to the weighted sum of position and confidence losses, which can be expressed by the following formula:

(1) $$L(x,c,l,g)=\frac{1}{N}(L_{conf}(x,c)+\alpha L_{loc}(x,l,g))$$

where l represents the detection box; g represents the real box; c represents the confidence of multi-class object; N represents the number of detection boxes that can effectively match the real box; $L_{conf}$ is the confidence loss; $L_{loc}$ is the position loss; α is the weight coefficient of position loss and confidence loss, which is set to 1 through cross validation.

Position loss is obtained by calculating the Smooth L1 loss between the detection and the real boxes. The offset of the coordinate center point (x,y), width w, and height h of the bounding box are regressed to obtain the minimum value of position loss. The relevant formula is as follows:

(2) $$L_{loc}(x,l,g)=\sum_{i\in Pos}^{N}\sum_{m\in \left\{cx,cy,w,h\right\}}x_{ij}^{k}\cdot smooth_{L1}(l_{i}^{m}-{\overset{⌢}{g}}_{j}^{m})$$

where Pos represents the aggregate of all positive samples; $x_{ij}^{k}$ indicates whether the object category *k* predicted by the *i*-th detection box is consistent with the classification label of the *j*-th real box, 1 if consistent, 0 otherwise; $l_{i}^{m}$ represents the coordinates of the *i*-th detection box; $g_{j}^{m}$ represents the coordinates of the *j*-th real box.

(3) $$\begin{array}{c} {\overset{⌢}{g}}_{j}^{cx}=(g_{j}^{cx}-d_{i}^{cx})/d_{i}^{w}\\ {\overset{⌢}{g}}_{j}^{cy}=(g_{j}^{cy}-d_{i}^{cy})/d_{i}^{h}\\ {\overset{⌢}{g}}_{j}^{w}=\log(g_{j}^{w}/d_{i}^{w})\\ {\overset{⌢}{g}}_{j}^{h}=\log(g_{j}^{h}/d_{i}^{h}) \end{array}$$

where $g_{j}^{cx}$ and $g_{j}^{cy}$ represent the coordinate center points of the *j*-th real box; $g_{j}^{w}$ and $g_{j}^{h}$ represent the width and height of the *j*-th real box, respectively; $d_{i}^{cx}$ and $d_{i}^{cy}$ represent the coordinate center points of the *i*-th detection box, respectively; $d_{i}^{w}$ and $d_{i}^{h}$ represent the width and height of the *i*-th detection box, respectively.

Confidence loss is obtained by calculating the Softmax loss of the confidence of the multi-class object, which is expressed by the following formula:

(4) $$L_{conf}(x,c)=-\sum_{i\in Pos}^{N}x_{ij}^{p}\log({\overset{⌢}{c}}_{i}^{p})-\sum_{i\in Neg}\log({\overset{⌢}{c}}_{i}^{0})$$

(5) $${\overset{⌢}{c}}_{i}^{p}=\exp(c_{i}^{p})/\sum_{p}\exp(c_{i}^{p})$$

where p represents the object category; $x_{ij}^{p}$ indicates whether the object category p predicted by the *i*-th detection box is consistent with the classification label of the *j*-th real box; ${\overset{⌢}{c}}_{i}^{p}$ represents the probability that the object category predicted by the *i*-th detection box is p, if the match is correct, then the loss is small when the probability is great; ${\overset{⌢}{c}}_{i}^{0}$ represents the probability that the object category predicted by the *i*-th detection box is background, if no object is present in the detection box, then the loss is small when the probability is great.

### 2.2. Shortcomings of SSD in Vehicle Detection

SSD absorbs the advantages of Faster R-CNN and YOLO. However, the SSD network model still has many disadvantages when it is applied to vehicle detection, including the unsatisfactory detection effect for small-scale vehicles, low detection precision under bad weather conditions, and easy missing detection of blocked vehicles. The analysis and summary reasons are as follows:

(1) In the front view of a smart car, the long-distance vehicle object only accounts for a small proportion of the image area in the collected detection image, and the vehicle object scale is small. Although the SSD network model has a multi-scale feature extraction network, the SSD adopts a nondiscriminatory method for different scale features, and simply selects a few feature layers for prediction without considering that the shallow and deep convolutional layers contain different local details and textural and semantic features. Therefore, the SSD network model has insufficient ability to extract features of small-scale vehicle objects and has yet achieved a satisfactory detection effect.
(2) In the actual road scenes, different vehicle objects have obvious differences in characteristics, such as color, shape, and taillights, and are easily affected by changes in lighting conditions, severe weather interference, and road object occlusion. These conditions bring many challenges to the accurate detection of front vehicles. The original SSD network model has poor vehicle detection performance in complicated environments, and its robustness and environmental adaptability are poor.
(3) In the network training process, the regression task is only for matching the correct detection box. Accordingly, the corresponding loss will be directly set to zero when no vehicle object is present in some pictures of the dataset; thus, the other pictures are not fully utilized. In the ranking of confidence scores, the number of negative detection boxes is much larger than that of positive detection boxes. Accordingly, the training network pays great attention to the proportion of negative samples, thereby resulting in the slow training speed of the network model.
(4) When the smart car passes through intersections, urban arterial roads, and traffic jam areas, a single detection image collected may include multiple vehicle objects, thereby inevitably resulting in mutual occlusion between vehicle objects. However, the original SSD network model has poor detection performance for overlapping objects, and it is prone to miss detection in multi-object scenes.

## 3. Improved SSD Network Model

### 3.1. Improved Basic Structure of SSD

Considering the limited feature extraction ability of the original SSD network model for small-scale vehicle objects, the model structure needs to be reasonably improved. The direct way to enhance the feature extraction ability is to expand the network depth by adding multiple convolutional layers. However, this method will lead to the rapid increase in the network model parameters, which is prone to over-fitting phenomenon and greatly reduces the detection efficiency of the training network. In recent years, the local topology represented by the inception block gradually shines in the field of object detection with the rapid development of deep learning and convolutional neural network. Inception block was first proposed by Szegedy at the International Conference on Computer Vision and Pattern Recognition (CVPR) in 2015, which was successfully applied in GoogLeNet and achieved excellent classification and recognition results in the ILSVRC2014 (Imagenet Large Scale Visual Recognition Challenge 2014) [36,37]. Inception block is a small network structure added to the network model. The convolutional kernels of different sizes are used to extract features of the same input layer, thereby greatly expanding the overall width of the network. This approach is helpful in enhancing the feature extraction ability of the network model and to avoid over-fitting phenomenon.

SSD creates a multi-scale feature extraction network in the form of a feature pyramid by adding multiple sets of convolutional layers behind the basic network. The shallow and high-level feature maps are responsible for feature learning and prediction of small-scale and large-scale objects, respectively. The shallow-level feature maps contain detailed information, but the semantic features are insufficient. The high-level feature maps are the opposite. Each feature layer in the original SSD only relies on a single feature input from the previous layer, which cannot achieve context information sharing during multi-scale feature extraction, thereby greatly affecting the detection performance of the network model. Feature fusion is an effective approach to solve this problem. Feature fusion is to process feature layers of different scales and form a new feature layer. The fusion of high-level semantic features and shallow detail information helps strengthen the connection between feature layers and realize context information sharing in the network model.

**Figure 2:**
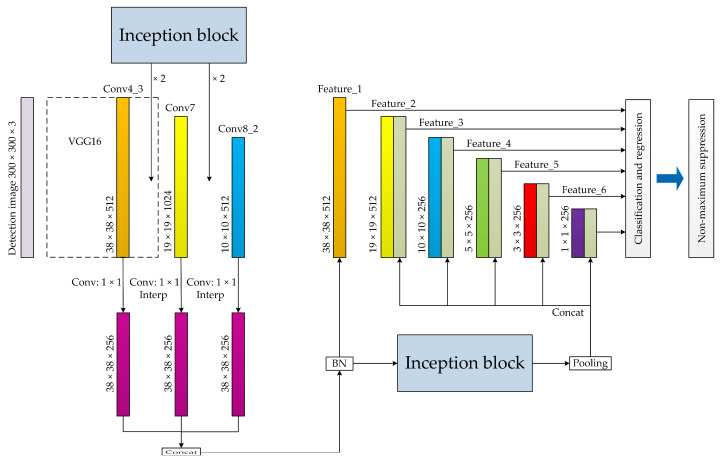
The basic structure of the improved SSD network model.

**Figure 3:**
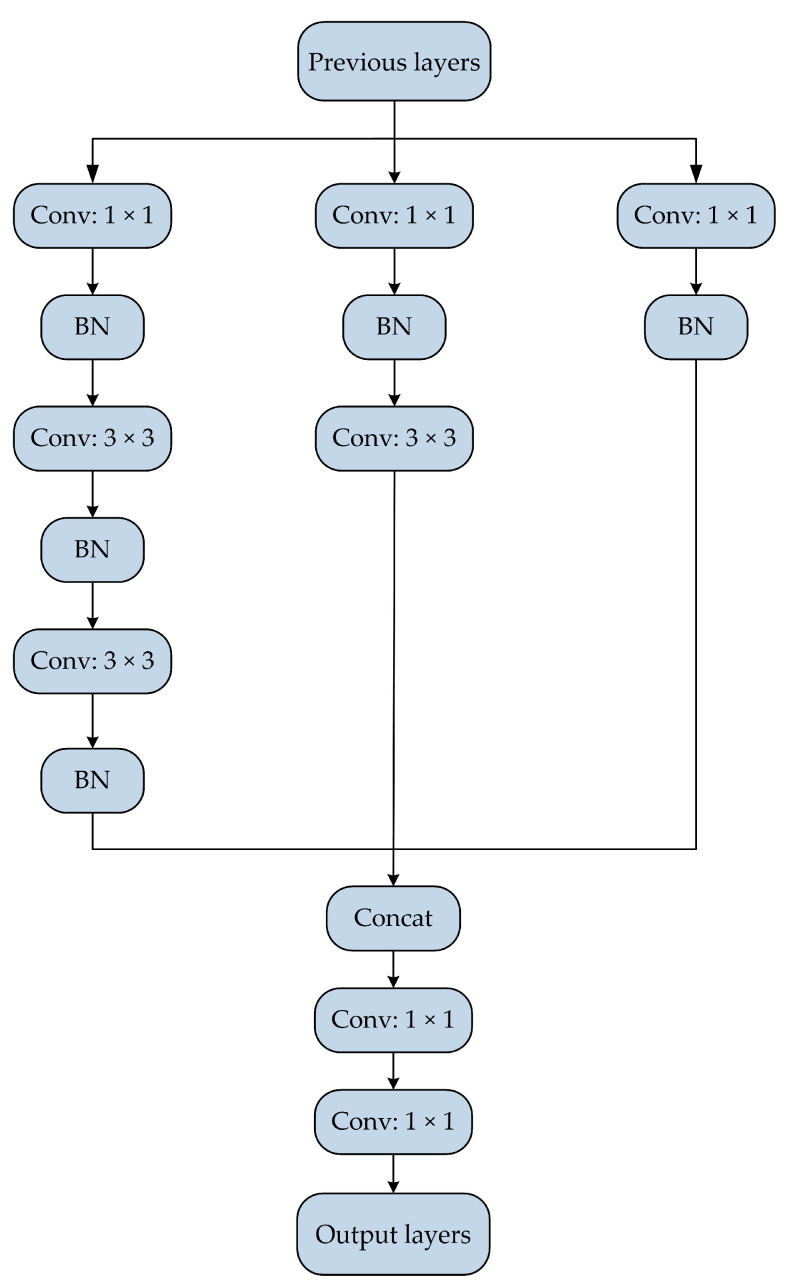
The internal structure of the inception block.

Aiming at the problem that the original SSD network model has insufficient ability to extract the features of small-scale vehicle objects in complicated environments, this study extends and deepens the neural network and improves the basic structure of SSD by combining inception block and feature fusion. Figure 2 shows the basic structure of the improved SSD network model, and Figure 3 presents the internal structure of the inception block.

Figure 2 shows that the inception block has been used several times in the improved SSD network model. First, four groups of inception blocks are added to the basic network of SSD to extract the local features of the network. The newly created interp layers performs feature layer scale conversion on the Conv7 and Conv8_2 layers through bilinear interpolation, and the output scale is 38 × 38, thereby making it the same size as the Con4_3 layer. Then, the newly created concat layer combines the above-mentioned three feature layers with the same scale into a new feature layer through the concatenation operation to achieve feature fusion. This specific feature layer contains context information and is used as Feature_1 to construct a new multi-scale feature extraction network after batch normalization (BN) processing. Finally, the convolutional kernel of size 3 × 3 is used to reduce the feature layer scale of the network layer by layer with Feature_1 as the base layer. Five feature layers with different scales are generated. A group of inception blocks is again added, and five new feature layers corresponding to the above-mentioned five feature layers are obtained by pooling the Feature_1_inception layer. A new concat layer is again created, and the concatenation operation is conducted to fuse the five groups of feature layers with the same scale one by one to form Feature_2, Feature_3, Feature_4, Feature_5, and Feature_6. A new multi-scale feature extraction network is established by combining the aforementioned layers with Feature_1. The new multi-scale feature extraction network can reuse the key features, which is conducive to improving the overall feature extraction ability of the network model.

Figure 3 shows that the inception block mainly uses convolutional kernels of 5 × 5, 3 × 3, and 1 × 1 to perform convolution operation on the input features, and two 3 × 3 convolutional layers in series are used instead of 5 × 5 convolutional layers. The advantage of this structural design is that it can further reduce the parameters of the model while keeping the original receptive field unchanged. The feature extraction ability of the inception block can be improved by introducing nonlinear transformations. In the internal structure of the inception block, the ratio of the number of convolutional kernels of 5 × 5, 3 × 3, and 1 × 1 is 1:2:1. The 1 × 1 convolutional layer is added in front of the 5 × 5 and 3 × 3 convolutional layers to reduce the number of input feature channels and the overall calculation. At the end of the structure, two 1 × 1 convolutional layers are added after the concat layer to further enhance the nonlinear computing ability of the inception block.

The network model can extract the features of the hidden layers in the network to the greatest extent and fully share the context information by using the inception block and feature fusion. This approach helps in enhancing the feature extraction ability for small-scale vehicle objects in complicated environments. Although the improved SSD network model increases the structural complexity and the number of parameters, it does not have a significant impact on the computational load because the scale of the feature layer is kept in a small range, and BN processing is used several times. It can ensure that the model has a fast training speed and good real-time detection performance while improving the level of feature extraction.

### 3.2. Weighted Mask

In the original SSD training network, when a picture has no vehicle objects in the dataset, the corresponding classification loss function will be directly set to zero, and the remaining valuable images in the dataset cannot be fully utilized. Considering that the number of negative detection boxes is much larger than that of positive detection boxes, the detection boxes with high confidence scores are used. The ratio of positive and negative samples is controlled to 1:3, which undoubtedly reduces the convergence speed of the training network.

On the basis of the shortcomings of the original SSD network model during training, this paper calculates the weighted mask for sample classification and regression tasks when using relevant datasets for training. The calculation method of weighted mask is as follows:

(1) When K detection boxes are present, the number of positive samples is N, the number of negative samples is M, K=N+M, and the classification label label is set.
(2) When N>0 the weighted mask for positive sample classification is set to pos_mask=label/N.
(3) When M>0 and the ratio of positive and negative samples is controlled to 1:3, the weighted mask for negative sample classification is set to $neg\_mask=\left\{1-label\right\}/M\times 3$.
(4) The weighted mask used for classification task is cls_mask=pos_mask+neg_mask
(5) Assuming that the weight coefficient of regression task is α the weighted mask used for regression task is reg_mask=pos_mask×α.

This study ensures that the training network pays great attention to the sample data with high classification difficulty by using weighted mask in the training process. This approach is beneficial to solve the problem of the imbalance between the background and the positive and negative sample data and further accelerate the training speed of the network model.

### 3.3. Improved Loss Function

The original SSD network model has a good detection effect on a single-vehicle object in simple environments. However, this model cannot achieve satisfactory detection results when detecting many vehicle objects in the multi-object scenes or vehicle objects with severe occlusion. The missing detection, false detection, and inaccurate object positioning easily appear.

Considering the above-mentioned deficiencies, this study improves the loss function and adds exclusion loss on the basis of the original position and confidence losses. The improved loss function can be expressed by the following formula:

(6) $$L=L(x,c,l,g)+\gamma L_{RepGT}$$

where $L_{RepGT}$ is the exclusion loss, and γ is the weight coefficient, which is used to balance the auxiliary loss.

This study allows $P_{+}=\left\{P\right\}$ to represent the aggregate of all candidate boxes with *IoU* greater than 0.5, and $G_{+}=\left\{G\right\}$ to represent the aggregate of all real boxes. In any candidate box $P\in P_{+}$, this study allows the real box with a large *IOU* as its specified object, namely:

(7) $$G_{Attr}^{P}=\underset{G\in G_{+}}{\arg\;\max}IoU(G,P)$$

Given that the exclusion loss aims to make the candidate box repel the adjacent real box, the exclusion object for any candidate box $P\in P_{+}$ is the real box with a large *IoU* except the specified object, namely:

(8) $$G_{Rep}^{P}=\underset{G\in G_{+}\left\{G_{Attr}^{P}\right\}}{\arg\;\max}IoU(G,P)$$

This study allows $B^{P}$ to be the detection box regressed from candidate box P. The overlapping IoG between $B^{P}$ and $G_{Rep}^{P}$ can be expressed by the following formula:

(9) $$IoG(B^{P},G_{Rep}^{P})=\frac{area(B^{P}\cap G_{Rep}^{P})}{area(G_{Rep}^{P})}$$

The exclusion loss can be calculated by the following formula:

(10) $$L_{RepGT}=\frac{IoG(B^{P},G_{Rep}^{P})}{\left|P_{+}\right|}$$

The exclusion loss is used to increase the distance between the detection box and the surrounding nonvehicle objects. If an overlap area with the surrounding nonvehicle objects is observed, then the detection box will be subject to additional penalties. The penalty will be great when the overlap area is large, and vice versa. Therefore, adding exclusion loss on the basis of the original loss function can prevent the detection box from moving to adjacent nonvehicle objects. This approach is helpful in accurately locating vehicle objects and effectively improving the detection performance for overlapping objects in multi-object scenes.

## 4. Vehicle Detection Experiments and Discussion

### 4.1. Experimental Environment

The software environment is as follows: Windows 10 64-bit operating system, TensorFlow deep learning framework, CUDA 9.1, cuDNN 7.1, Python 3.7.0, and MATLAB R2018a.

The hardware environment is as follows: Intel (R) Core (TM) i7-7700 CPU@3.60 GHz processor, 32 GB memory, and NVIDIA GeForce GTX 1080Ti GPU, 11 GB.

### 4.2. Vehicle Detection Experiment Based on KITTI Dataset

#### 4.2.1. KITTI Dataset

This article uses the KITTI vision benchmark suite for vehicle detection experiments, which was jointly developed by Karlsruhe Institute of Technology and Toyota Technological Institute at Chicago. This dataset has now become an internationally used algorithm evaluation dataset for autonomous driving scenarios. KITTI dataset mainly focuses on performance evaluation of various computer vision technologies, including optical flow, stereo image, visual ranging, and object detection [38,39]. This dataset covers real road images in several scenarios, such as cities, villages, and highways. Each sample image contains up to 15 vehicle objects and 30 pedestrian objects, and the image size is 1242 × 375 pixels. The whole dataset is composed of video streams collected with binocular cameras, and it can be divided into five categories: road, city, residential, campus, and person.

**Figure 4:**
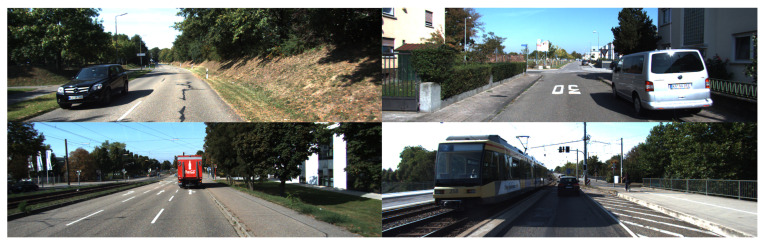
The example image of the KITTI dataset.

KITTI dataset includes label data and does not require manual annotation, thereby providing reliable image content information for model training. Considering that 7481 sample images with corresponding label files are present in the dataset, 5985 images are divided as the training set, and 1496 images are divided as the testing set. The ratio of the training set to testing set is 4:1. The sample images can be divided into eight categories according to the object classification of annotation information: car, van, truck, pedestrian, pedestrian (sitting), cyclist, tram, and misc or “dontcare”. During the data preparation, all label files need to be converted from txt format to XML format required for SSD training. This work only retains car, van, truck, and tram and eliminates other irrelevant categories due to the focus on vehicle object detection. Figure 4 presents the example image of the KITTI dataset.

#### 4.2.2. Network Training and Evaluation Indexes

**Figure 5:**
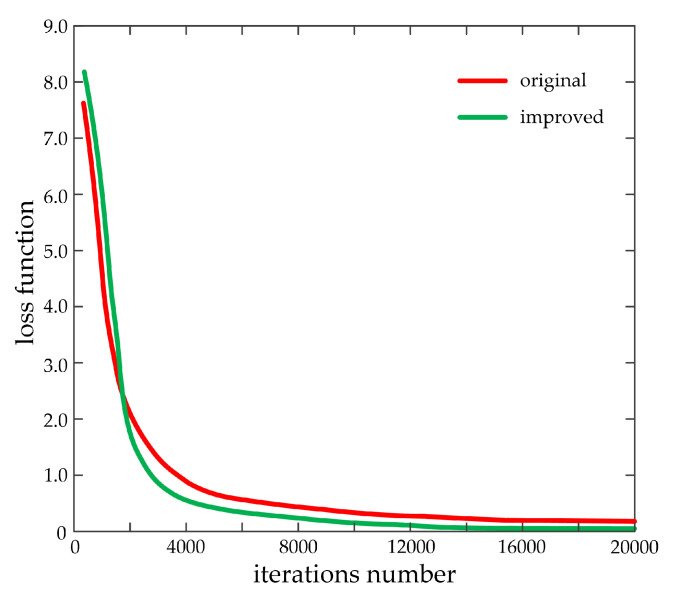
The loss functions of SSD before and after improvement.

In this study, the stochastic gradient descent method is used for optimization. The weight parameters of the training network are continuously updated by using the back propagation algorithm. The initial learning rate is set to 0.001, the momentum factor is set to 0.9, and the weight attenuation factor is set to 0.0005. The size of the learning rate is closely related to the convergence speed of the training network. If the setting is large, then the network model will not converge. By contrast, if the setting is small, then the convergence speed will be slowed down. In this study, the maximum number of iterations of the training network is 20,000. The learning rate is set to 0.001 in the first 12,000 times, 0.0001 from 12,000 to 16,000 times, and 0.00001 after 16,000 times. The L2 regularization is used for the loss function to prevent overlearning the features of the training set and avoid the occurrence of over-fitting. Figure 5 shows the loss functions of SSD before and after improvement.

The aforementioned figure demonstrates that the improved loss function is slightly larger than that of the original at the beginning of training. This condition may be due to the improvement of the loss function and the addition of exclusion loss. However, the improved loss function value is quickly lower than that of the original with the continuous iteration of the training network and finally gradually decreases to zero, thereby reflecting the advantage of using weighted mask. As the number of iterations is 3400, the distance between the two loss functions reaches the maximum. As the maximum number of iterations reaches 20,000, the distance between the two loss functions achieves the minimum. In summary, the convergence speed of the improved SSD network model is higher, thereby indicating that the problem of sample data imbalance has been effectively solved.

**Figure 6:**
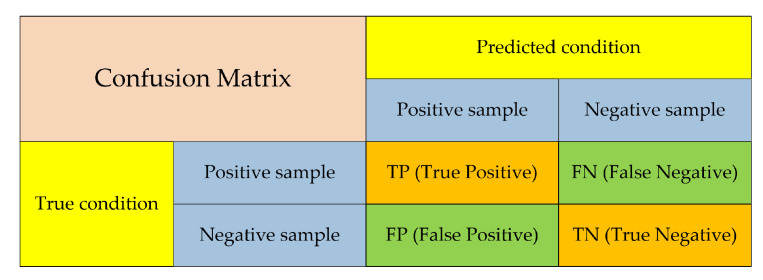
Confusion matrix.

In the vehicle detection algorithm, evaluation indexes must be used to accurately evaluate the detection performance. Considering that the detection image includes positive and negative samples, four prediction cases are present for the detection result, and the confusion matrix is shown in Figure 6.

The evaluation indexes, such as precision, recall and mean average precision (mAP), can be calculated according to the confusion matrix.

In this article, precision refers to the proportion of samples whose detection results are the vehicle objects that are correctly detected, and it can be expressed as follows:

(11) $$P=\frac{TP}{TP+FP}$$

Recall refers to the proportion of vehicle objects that are correctly detected, and it can be expressed as follows:

(12) $$R=\frac{TP}{TP+FN}$$

mAP is one of the important evaluation indexes of object detection algorithms, and it can be expressed as follows:

(13) $$mAP=\frac{\sum AP}{N}=\frac{\sum \int_{0}^{1}P(R)dR}{N}$$

where *N* is the category number of the objects.

#### 4.2.3. Experimental Test Results and Analysis

**Figure 7:**
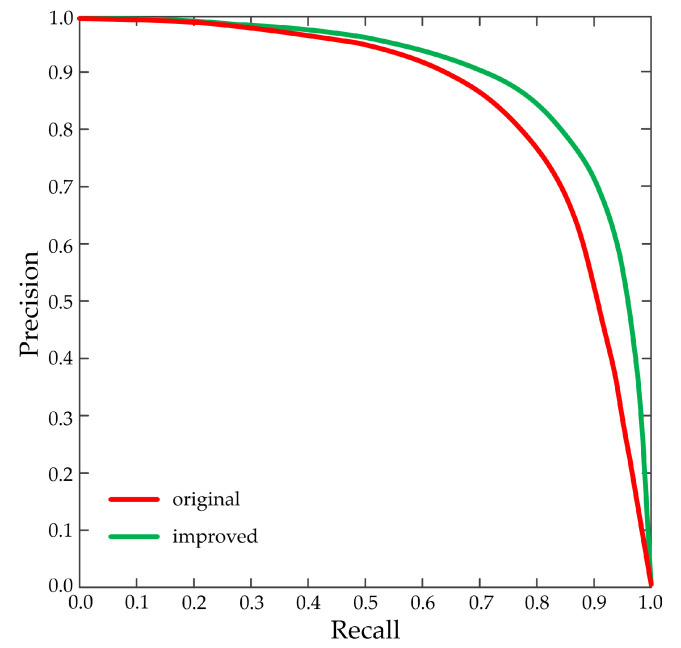
The precision-recall curves about the original and improved SSD.

In the NMS algorithm, the *IoU* threshold needs to be manually set. Different *IoU* thresholds will produce diverse precision and recall, and the setting of the *IoU* threshold is closely related to the detection performance of the network model. After repeated experimental tests, the *IoU* threshold is set to 0.5. Figure 7 shows the precision-recall curves about the original and improved SSD. The P–R curve uses recall and precision as the horizontal and vertical coordinates, respectively, which is a common curve used to measure the performance of the detection algorithm. The corresponding recall is low when the precision is high. When precision is at a high value, the probability of false detection is low. When recall is at a high value, the probability of missing detection is low.

The above-mentioned figure demonstrates that the improved P–R curve is more inclined to the upper right corner than the original one, thereby indicating that the detection performance of the improved SSD is better than the original one. When the recall is 90%, the improved precision is 70%, while that of the original is 50%. The area enclosed by the P–R curve and two coordinate axes of the improved is larger than that of the original, thereby reflecting that the improved SSD has obvious advantages in average detection precision.

**Figure 8:**
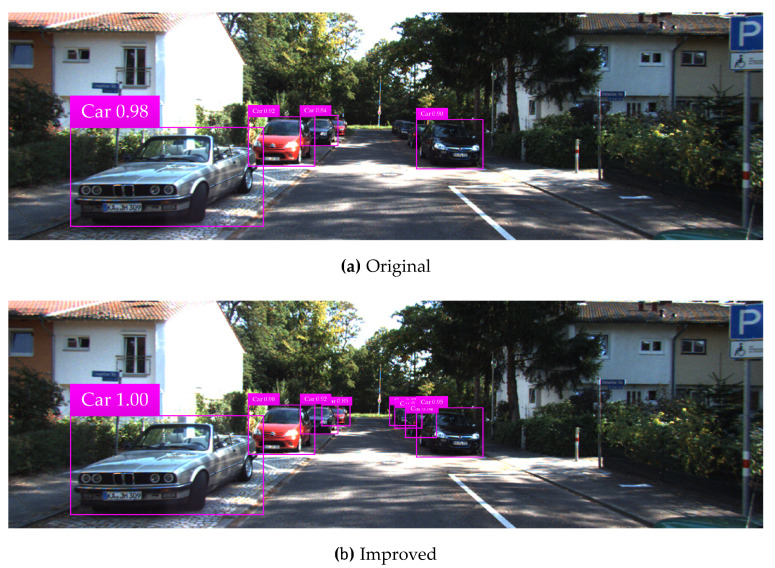
Vehicle detection test results in the shadow environment based on the original and improved SSD.

**Figure 9:**
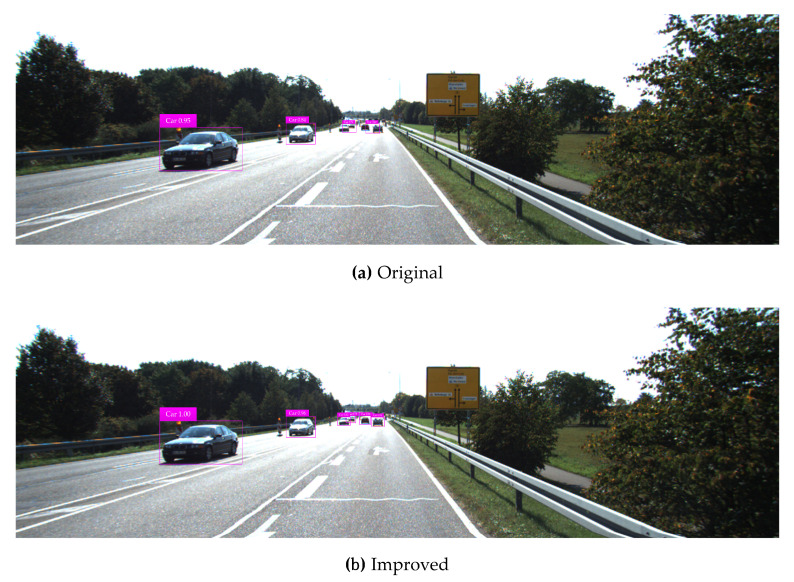
Vehicle detection test results for multi-scale objects based on the original and improved SSD.

**Figure 10:**
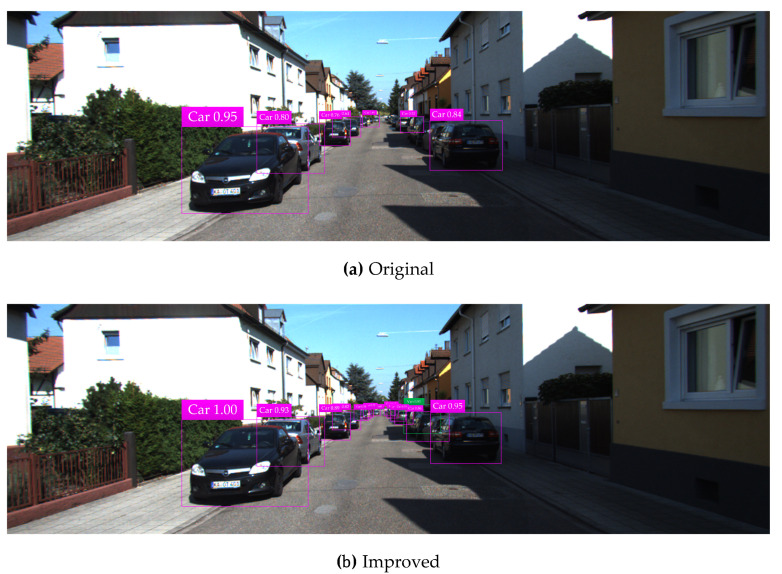
Vehicle detection test results under occlusion based on the original and improved SSD.

**Figure 11:**
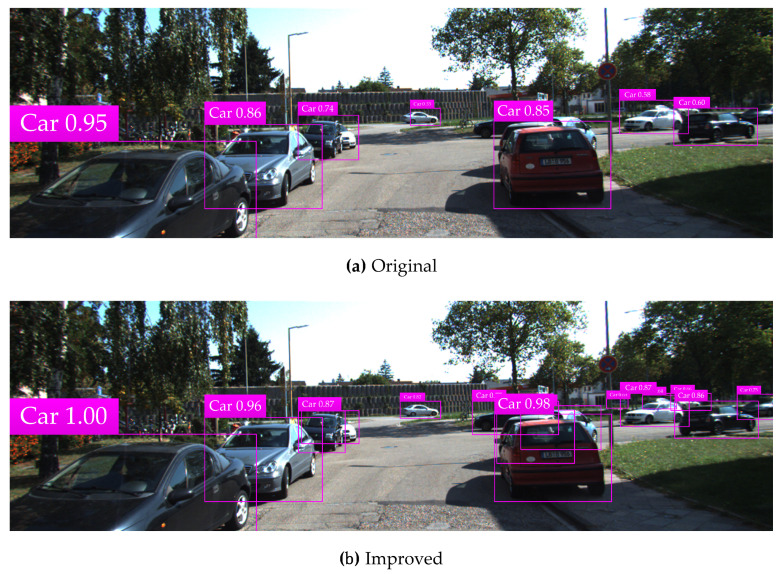
Vehicle detection test results at the road intersection based on the original and improved SSD.

**Figure 12:**
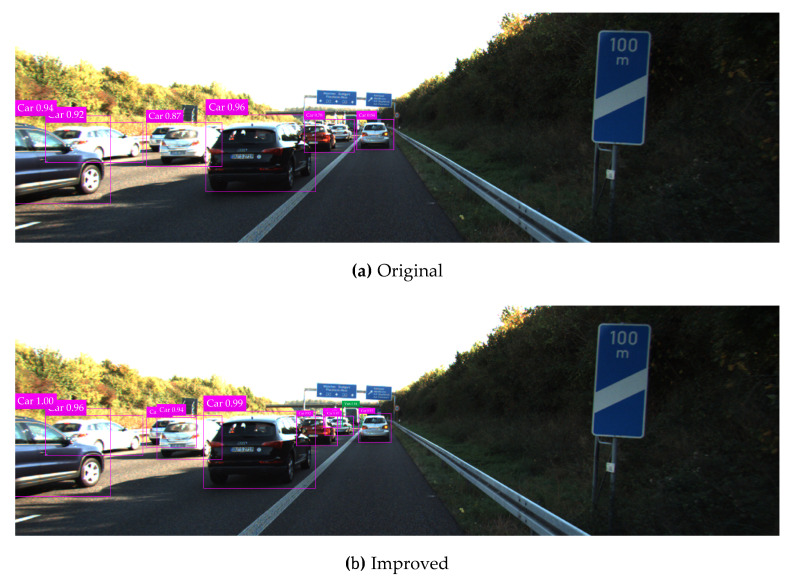
Vehicle detection test results in the traffic jam environment based on the original and improved SSD.

The KITTI testing set is used for vehicle detection test, and test results in various complicated environments are illustrated in Figure 8, Figure 9, Figure 10, Figure 11 and Figure 12. Among them, panels (a) and (b) are carried out based on the original and improved SSD, respectively. Figure 8 shows that in the shadow environment, the original SSD only detects four vehicle objects at a short distance, while the others at a long distance are missed. However, the improved SSD achieves the detection of all vehicle objects, and the confidence scores have been enhanced to a certain extent. Figure 9 indicates that the original SSD has the cases of missing detection and inaccurate positioning for small-scale vehicle objects, and the improved SSD achieves valid detection and accurate positioning of multi-scale vehicle objects. Figure 10 shows that multiple vehicle objects are blocked to varying degrees, and the original SSD causes a great deal of missing detection on the vehicle objects that are heavily blocked, and inaccurate positioning for vehicle objects at a long distance can be observed. By contrast, the improved SSD achieves valid detection of all cars and vans. Figure 11 shows that the vehicle objects are located at the road intersection, which is the typical traffic accident prone area. The original SSD only detects the vehicle objects with obvious feature information, and the improved SSD correctly detects all vehicle objects and effectively improves the corresponding confidence scores. Figure 12 demonstrates that in the traffic jam environment, the object density is high and mostly back-viewed. The original SSD causes missing detection on the vehicle objects in the far-field of view, and the improved SSD still realizes valid detection of all vehicle objects.

The vehicle detection test results show that the detection performance of the improved SSD network has laudable advantages, which is mainly attributed to the improvement of SSD basic structure and loss function. The proposed vehicle detection algorithm has excellent robustness and environmental adaptability for complicated traffic environments and road scenes, and the detection precision has been further improved.

### 4.3. Vehicle Detection Based on Self-Made Vehicle Dataset

This study also conducts a performance test on the basis of a self-made vehicle dataset to fully check the comprehensive detection performance of the proposed algorithm, in addition to the public KITTI dataset. The self-made vehicle dataset mainly comes from the vehicle collection images in the actual road scenes, including five types of weather, such as sunny, cloudy, rainy, snowy, and mild smoggy days, which can fully display various weather conditions that may be encountered. The training samples in the self-made vehicle dataset are labeled by the Ground Truth Labeler toolbox in MATLAB software, and negative samples of nonvehicle objects are added. The training set contains 4500 images, the testing set contains 1500 images, and the ratio of the training set to testing set is 3:1. The original and improved SSD network models are used to carry out vehicle detection experiments. After data statistics and classification, the vehicle detection test results under different weather conditions are illustrated in Table 2.

The aforementioned table demonstrates that the vehicle detection precision of the improved SSD network model is higher than that of the original network model under the same weather conditions. In the original mAP, the detection precision in sunny days is highest, reaching 91.56%, and the detection precision in mild smoggy days is lowest, reaching 80.21%. In the improved mAP, the detection precision in sunny days is highest, reaching 95.78%, and the detection precision in mild smoggy days is lowest, reaching 85.10%. In summary, the mAP of the improved is 91.76%, and that of the original is 86.70%. Test results show that the vehicle detection precision is high when the weather visibility is also high, and vice versa. The proposed detection algorithm can adapt to different weather conditions and still has high accuracy under bad weather conditions, thereby reflecting strong anti-jamming capabilities, which can be well applied to the front vehicle detection for autonomous vehicles.

### 4.4. Discussion

The proposed algorithm is compared with other methods to check the technical level of vehicle detection algorithm. Table 3 illustrates the performance comparison statistics of algorithms on the basis of the KITTI dataset.

The aforementioned table demonstrates that all algorithms conducted the vehicle detection experiments on the basis of the KITTI dataset. The mAP value and average processing time per frame are the main evaluation indexes for performance comparison. Reference [40] adopted a deep network and encoder named Pointpillars for object detection, which can be used for end-to-end training on LiDAR point clouds. This method had a fast detection speed for vehicle detection, but its mAP value was the lowest among all detection algorithms, and the accuracy needed to be improved. Reference [41] utilized a unified deep neural network called MS-CNN, which consisted of proposal and detection sub-networks. Multi-scale object detection was realized by feedforwarding a single input image through the network. This method achieved high vehicle detection precision. However, the average processing time was long to meet the real-time requirements of smart cars for vehicle detection. Reference [42] proposed a cascade object detection system on the basis of a two-stage regression, which can achieve rapid detection of vehicle objects, by referring to the advantages of two-stage and one-stage detection methods. In comparison with reference [41], the detection precision and speed obtained by this method were improved by different ranges and can still be further enhanced. The average processing time of the original SSD network model is comparatively short. However, the mAP value is still relatively low, thereby resulting in missing or false detection in complicated environments. In comparison with the above-mentioned algorithms, the comprehensive detection performance of the proposed algorithm is the best. The AP values in easy, moderate and hard modes are all the highest, and the mAP value is the largest, reaching 92.18%, and the average processing time per frame is the shortest, reaching 15 ms. Compared with the existing deep learning-based detection methods, the improved SSD network model enables the proposed algorithm to obtain accuracy and real-time performance simultaneously, which is conducive to the realization of fast and accurate automatic vehicle detection. This is of great significance to ensure the accurate and efficient operation of smart cars in the real traffic scenes, which helps to vastly reduce the incidence of traffic accidents and fully protect people’s lives and property.

## 5. Conclusions

In this article, a front vehicle detection algorithm for smart car based on improved SSD model is proposed. First, the SSD network model is briefly introduced, and its problems and shortcomings in vehicle detection are analyzed and summarized. Then, targeted improvements are performed to the SSD network model, including major advancements to the basic structure of the SSD model, the use of weighted mask in network training, and enhancement to the loss function. Finally, vehicle detection experiments are carried out on the basis of the KITTI and self-made vehicle datasets to observe the algorithm performance in different complicated environments and weather conditions. The test results based on the KITTI dataset show that the mAP value reaches 92.18%, and the average processing time per frame is 15 ms. Compared with the existing deep learning-based detection methods, the proposed algorithm can obtain accuracy and real-time performance simultaneously, which is conducive to the realization of fast and accurate automatic vehicle detection. Meanwhile, the algorithm has excellent robustness and environmental adaptability for complicated traffic environments and anti-jamming capabilities for bad weather conditions.

In terms of the accuracy rate and working efficiency, the proposed vehicle detection algorithm has outstanding performance advantages, which is of great significance to ensure the accurate and efficient operation of smart cars in the real traffic scenes, and is beneficial to vastly reduce the incidence of traffic accidents and fully protect people’s lives and property. In the future, we can continue to focus on vehicle detection algorithms under extreme conditions and FPGA implementation of algorithms to further promote the comprehensive performance and practical meaning of the algorithm.

## Figures and Tables

**Table 1:** The main parameters of the SSD network model.

Layer	Convolutional Kernel Size	Convolutional Kernel Number	Step Size	Filling	Feature Map Size
Conv1_1	3 × 3	64	1	1	300 × 300
Conv1_2	3 × 3	64	1	1	300 × 300
Maxpool1	2 × 2	1	2	0	150 × 150
Conv2_1	3 × 3	128	1	1	150 × 150
Conv2_2	3 × 3	128	1	1	150 × 150
Maxpool2	2 × 2	1	2	0	75 × 75
Conv3_1	3 × 3	256	1	1	75 × 75
Conv3_2	3 × 3	256	1	1	75 × 75
Conv3_3	3 × 3	256	1	1	75 × 75
Maxpool3	2 × 2	1	2	0	38 × 38
Conv4_1	3 × 3	512	1	1	38 × 38
Conv4_2	3 × 3	512	1	1	38 × 38
Conv4_3	3 × 3	512	1	1	38 × 38
Maxpool4	2 × 2	1	2	0	19 × 19
Conv5_1	3 × 3	512	1	1	19 × 19
Conv5_2	3 × 3	512	1	1	19 × 19
Conv5_3	3 × 3	512	1	1	19 × 19
Maxpool5	3 × 3	1	1	1	19 × 19
Conv6	3 × 3	1024	1	1	19 × 19
Conv7	1 × 1	1024	1	0	19 × 19
Conv8_1	1 × 1	256	1	0	19 × 19
Conv8_2	3 × 3	512	2	1	10 × 10
Conv9_1	1 × 1	128	1	0	10 × 10
Conv9_2	3 × 3	256	2	1	5 × 5
Conv10_1	1 × 1	128	1	0	5 × 5
Conv10_2	3 × 3	256	1	0	3 × 3
Conv11_1	1 × 1	128	1	0	3 × 3
Conv11_2	3 × 3	256	1	0	1 × 1

**Table 2:** The vehicle detection test results under different weather conditions.

Sequence Number	Weather Condition	Original mAP (%)	Improved mAP (%)
1	Sunny	91.56	95.78
2	Cloudy	88.72	93.66
3	Rainy	86.65	92.25
4	Snowy	86.34	92.02
5	Mild Smoggy	80.21	85.10
Total	-	86.70	91.76

**Table 3:** The performance comparison statistics of algorithms on the basis of the KITTI dataset.

Sequence Number	Method	Easy	Moderate	Hard	mAP (%)	Average Processing Time (ms)/Frame	System Environment
1	Pointpillars [40]	88.35	86.10	79.83	84.76	16	Intel i7 CPU and 1080Ti GPU
2	MS-CNN [41]	90.03	89.02	76.11	85.05	400	Intel Xeon E5-2630 CPU@2.40 GHz; NVIDIA Titan GPU
3	HybridNet [42]	88.68	87.91	79.07	85.22	45	NVIDIA GTX 1080Ti GPU
4	Original SSD	90.67	89.56	82.39	87.54	28	Intel(R) Core(TM) i7-7700 CPU@3.60GHz; NVIDIA GeForce GTX 1080Ti GPU
Ours	Improved SSD	95.76	94.55	86.23	92.18	15	Intel(R) Core(TM) i7-7700 CPU@3.60GHz; NVIDIA GeForce GTX 1080Ti GPU
